# Predictors of bullying perpetration among adolescents attending secondary schools in Sokoto Metropolis, Nigeria, 2019

**DOI:** 10.11604/pamj.2021.39.49.22690

**Published:** 2021-05-19

**Authors:** Ismail Abdullateef Raji, Sulaiman Saidu Bashir, Adebowale Ayo Stephen, Chukwuma David Umeokonkwo, Mu'awiyyah Babale Sufiyan, Auwal Usman Abubakar, Kabir Sabitu

**Affiliations:** 1 Nigerian Field Epidemiology and Laboratory Training Program, 50 Haile Selassie Street, Asokoro, Abuja, Nigeria,; 2 Department of Community Medicine, Usmanu Danfodiyo University Teaching Hospital, Sokoto, Nigeria,; 3 Department of Community Medicine, Ahmadu Bello University, Zaria, Nigeria,; 4 Department of Epidemiology and Medical Statistics, University of Ibadan, Ibadan, Nigeria,; 5 Department of Community Medicine, Alex Ekwueme Federal University Teaching Hospital, Ebonyi, Nigeria

**Keywords:** Bullying, perpetration, adolescents, Sokoto, Nigeria

## Abstract

**Introduction:**

bullying affects up to 85% of in-school adolescents in Nigeria. It presents a potentially serious threat to healthy adolescent development. Bullying has not been extensively studied in Nigeria and more so in northern Nigeria. Therefore, we investigated the types and predictors of bullying perpetration among adolescents in secondary schools.

**Methods:**

we conducted a cross-sectional study between January and March 2019. Using a multistage sampling technique, we recruited 390 adolescents. We estimated the prevalence and types of bullying perpetration, and we examined the predictors of bullying among the participants using chi-square and binary logistic regression at a 5% level of significance.

**Results:**

the mean age of adolescents was 15.2 ± 1.9 years. Majority of the participants 234 (60.0%) were in late adolescence (15-19 years), and 205 (52.6%) were males. The most prevalent type of bullying perpetrated was verbal [69.7%; 95% CI = 64.9-74.3%]. Overall, 307 [78.7%; 95% CI = 74.3-82.6%] had perpetrated at least one type of bullying. Male gender (adjusted Odds Ratio (aOR): 2.70; 95%CI = 1.43 - 5.10), attending a boarding school (aOR: 7.93, 95% CI = 2.91 - 21.58) and frequent parental conflicts (aOR: 5.23, 95% CI = 2.15 - 12.71) were independent predictors of bullying perpetration.

**Conclusion:**

there is a high prevalence of bullying perpetration among adolescents in Sokoto metropolis, especially among males, those in boarding schools and those who experience frequent parental conflicts. We recommend that school principals should pay close attention to this behaviour and parents should be sensitized on the consequences of their domestic actions on their children.

## Introduction

Violence in schools affects about 240 million adolescents and children worldwide [1]. This violence denies them the fundamental human right to education [1]. Bullying, a form of violence, affects one out of every three teenagers globally [2]. It is considered to be the most prevalent form of violence in secondary schools [3]. Bullying behavior consists of three main types of participants: bullies (perpetrators), victims and bully-victims [2]. Bullying perpetration is a universal heart-rending problem affecting adolescents. It is recognized as an indicator of more serious violent behaviors, with its adverse effects on the victim and the perpetrator [4]. Bullying perpetration has been linked to a broad spectrum of adverse outcomes ranging from poor school results, mental health problems, to a vicious exhibition of bad behaviors that often persist into adulthood. Such unpleasant behaviors manifest in various forms of violence like intimate partner violence, gang violence, criminality and cultism [5-9]. This social menace has been widely reported among in-school children in several countries; however, variation exists in its prevalence between and within countries [10]. In North America, the prevalence of bullying perpetration ranged from 6.1% in Canada [11] to 10.6% in the United States (US) [12], while 57.8% of adolescents have perpetrated bullying in Australian schools [13]. In Asia and South America, the prevalence of bullying perpetration ranges between 9.0% in China [14] and 97.1% in India [15]. In sub-Saharan Africa, a high level of bullying among in-school children has been reported in Tanzania (25.0%), Malawi (44.5%) and Zambia (62.8%) [16-18]. Studies in different parts of Nigeria also found 85.0%, 33.1%, and 64.9% as prevalence of bullying in Benin city, Osun and Port Harcourt, respectively [19-21].

While a significant number of studies have been conducted in developed countries, not much information is available on bullying perpetration in Nigeria, particularly in Northern Nigeria. In Nigeria, unfortunately, parents and teachers tend to regard bullying as part of childhood experience which children must learn to tolerate as part of the process of growing up [22]. The lack of attention has allowed bullying to persist for a long time unchecked which could be responsible for the high prevalence of cultism in Nigerian Universities [23]. Research on bullying perpetration has not been given adequate attention in Sokoto, mainly because bullying is seen as a typical encounter in schools. Therefore, the knowledge obtained from this study could be used as an important motivational factor to develop school health programmes that would help curb the burden. Furthermore, the result of the study could provide an insight into the nature and extent of bullying, which can stimulate the formulation of legislation against bullying and violence in schools and possibly prevention programs which are presently rare in Nigeria. Therefore, this study sought to determine the prevalence and predictors of bullying perpetration among adolescents attending secondary schools in Sokoto metropolis.

## Methods

**Study area:** Sokoto is located in North-Western, Nigeria. The State metropolis has four local government areas (LGAs): Sokoto South, Sokoto North, Dange Shuni and Wamakko. The inhabitants are mainly Hausa and Fulani ethnic groups, but people from other ethnic groups in Nigeria are found in large numbers within the Metropolis. The educational system in Sokoto is the 9-3-4 system with nine years of basic education, three years of senior secondary and four years of higher education. Both public and private schools deliver this system of schooling, some with a mix of boys and girls and others with boys-only or girls-only. Also, some schools combine both Western and Islamic education, while others offer only Quranic education. Sokoto metropolis has 111 schools comprising 76 public and 35 private schools. Generally, bullying is not accepted by authorities in Sokoto metropolis; however, there are no specific provisions in the laws and policies of Sokoto state and Nigeria as a whole addressing specifically the phenomenon of bullying in schools. Hence, this is handled as part of other acts of violence generally.

**Study design and population:** a cross-sectional study was conducted among adolescents attending secondary schools in Sokoto metropolis between January 2019 and March 2019. An adolescent is any child between 10 and 19 years old. Adolescents who have spent less than a school term in their current school or not registered in the selected school (on an excursion or inter-school programs) but were present at the time of the study were excluded from the study.

**Sample size estimation and sampling technique:** the minimum sample size of 390 was obtained using the formula for calculating the sample size for descriptive [24]

$$n=\frac{Z\alpha^{2}pq}{d^{2}}$$

using a level of confidence (z) of 1.96, prevalence (p) of bullying perpetration of 0.649 from a previous study, [21] and a precision level (d) of 0.05. A multistage sampling technique was used to select the eligible respondents for the study. In stage one, a simple random sampling technique (balloting) was used to select two LGAs (Wamakko and Sokoto South) from the four metropolitan LGAs. In stage two, a list of schools in the selected LGAs was obtained from the state Ministry of Education. The schools were stratified into private (11 schools) and public (27 schools). Wamakko LGA had 18 schools (six private and 12 public) while Sokoto South had 20 schools (five private and 15 public). Two private schools and four public schools were selected using simple random sampling technique by balloting from each of the selected LGA, thus, giving a total of 12 schools (four private and eight public). In the final stage, students were stratified into junior and senior classes; a list of the students by class was obtained from each selected school to serve as the sampling frame. After that, a systematic sampling technique based on proportionate allocation was used to recruit study participants from each selected school to obtain the minimum sample size.

**Data collection and analysis:** a structured interviewer-administered questionnaire was used for data collection. The questionnaire consisted of questions adapted from previous studies [5,25-27]. Face and content validity of the questionnaire was done by senior colleagues who are experts in the field. We pretested the research instrument among 39 purposively sampled in-school adolescents (10% of sample size) from three randomly selected schools in one LGA (Dange Shuni) not selected for the study. The reliability coefficient, Cronbach's alpha, was 0.757.

**Definition of variables:** the dependent variable in this study was bullying perpetration. An adolescent was considered to have perpetrated bullying if he/she was involved in at least one of the following: physical bullying, verbal bullying, relational bullying and attack on the property of fellow adolescents. The independent variables include questions that addressed family factors, peer factors, school factors and sociodemographic profile.

**Method of analysis:** we used IBM SPSS® version 23.0 and Microsoft Excel version 2016 for data cleaning and analyses. Frequencies, proportions, mean, and standard deviation were used to describe the sociodemographic profile and types of bullying perpetration. Chi-square test was used to determine the association between bullying perpetration and sociodemographic profile, family factors, peer factors and school factors. A binary logistic regression model was used to identify factors that predict bullying perpetration. All statistical analyses were performed at a 5% level of significance. However, at the stage of bivariate analysis, variables that were significant at 10% were equally included in the multivariate analysis.

**Ethical approval:** we obtained ethical approval (reference number SMH/1580/V.IV) for this study from Sokoto State Ministry of Health research and ethics committee. We also got permission from the Sokoto State Ministry of Education (reference number EST/GEN/2457/Vol.1). We obtained written informed consent from students above 18 years, and assent from those below 18 years along with their parental permission. The anonymity and confidentiality of the information provided by the participants were assured.

## Results

**Figure 1:**
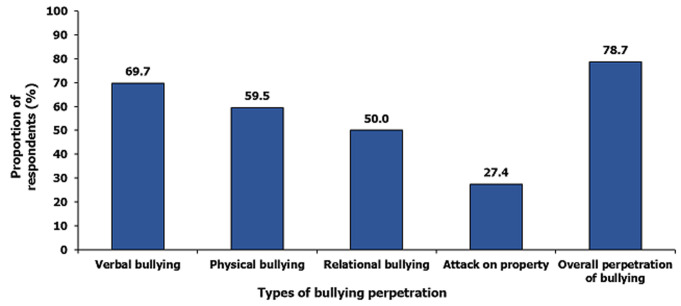
prevalence of bullying perpetration among in-school adolescents in Sokoto metropolis, 2019 (n= 390)

The mean age of the respondents was 15.2 ± 1.9 years, with more than half [234 (60.0%)] in the age group 15-19 years. About half, 185 (47.4%) were females and many 250 (64.1%) were brought up in a polygamous family setting. More than half, 241(61.8%) and 229(58.7%) was in the senior class and attending day school, respectively (Table 1). Overall, 307 (78.7%; 95% CI = 74.3-82.6%) of the respondents have perpetrated at least one type of bullying. Perpetration of verbal bullying (69.7%; 95%CI = 64.9-74.3%) was the commonest form while an attack on property (27.4%; 95%CI = 23.1-32.2%) was the least common form of bullying perpetrated (Figure 1). A higher proportion of bullying perpetration was observed among 15-19 years old, 192 (82.1%) compared to 10-14 years old, 115 (73.7%), and this was statistically significant, (p = 0.049). A higher proportion of males, 181(88.3%) compared to females, 126 (68.1%) were bullies, (p <0.001). Adolescents who often experience parents quarrel or fight, 99 (93.4%) were more likely to be bullies compared to those who did not, 208 (73.2%), p < 0.001 (Table 2). Males were more likely to bully others (aOR: 2.70; 95% CI = 1.43 - 5.10), and those in boarding school were more likely to be perpetrators of bullying (aOR: 7.93; 95% CI = 2.91 - 21.58). Respondents that often experience parental conflicts were more likely to be perpetrators of bullying (aOR: 5.23; 95% CI = 2.15 - 12.71) Table 3.

**Table 1:** socio-demographic profile of in-school adolescents in Sokoto metropolis, 2019 (n = 390)

Variables		Frequency	Percent (%)
**Age group (Years)**			
	10-14	156	40.0
	15-19	234	60.0
**Sex**			
	Female	185	47.4
	Male	205	52.6
**Religion**			
	Christian	105	26.9
	Muslim	285	73.1
**Ethnic group**			
	Hausa/Fulani	227	58.2
	Yoruba	105	26.9
	Ibo	58	14.9
**Family type**			
	Monogamous	140	35.9
	Polygamous	250	64.1
**School type**			
	Public	236	60.5
	Private	154	39.5
**Type of class**			
	Junior	149	38.2
	Senior	241	61.8
**Kind of school**			
	Boarding	161	41.3
	Day	229	58.7

**Table 2:** relationship between bullying perpetration and sociodemographic, family, and school factors among in-school adolescents in Sokoto metropolis, 2019, (n = 390)

Variables		Bullying perpetration		p value
		Yes	No	
**Age group (Years)**				
	10-14	115 (73.7)	41 (26.3)	0.049*
	15-19	192 (82.1)	42 (17.9)	
**Gender**				
	Male	181 (88.3)	24 (11.7)	< 0.001*
	Female	126 (68.1)	59 (31.9)	
**Religion**				
	Christian	94 (89.5)	11 (10.5)	0.002*
	Muslim	213 (74.7)	72 (25.3)	
**Ethnic group**				
			
	Hausa/Fulani	164 (72.2)	63 (27.8)	< 0.001*
	Yoruba/Ibo	143 (87.7)	20 (12.3)	
**Family type**				
	Monogamous	123 (87.9)	17 (12.1)	0.001*
	Polygamous	184 (73.6)	66 (26.4)	
**Public/private**				
	Public	200 (84.7)	36 (15.3)	< 0.001*
	Private	107 (69.5)	47 (30.5)	
**Class**				
	Junior class	119 (79.9)	30 (20.1)	0.663
	Senior class	188 (78.0)	53 (22.0)	
**Accommodation status**				
	Boarding	150 (93.2)	11 (6.8)	< 0.001*
	Day	157 (68.6)	72 (31.4)	
**Often experience parents quarrel or fight**				
	Yes	99 (93.4)	7 (6.6)	< 0.001*
	No	208 (73.2)	76 (26.8)	
**Parent monitor whereabouts and friends**				
	Yes	304 (79.2)	80 (20.8)	0.083**
	No	3 (50.0)	3 (50.0)	
**Get desired help from parents**				
	Yes	299 (78.5)	82 (21.5)	0.451
	No	8 (88.9)	1 (11.1)	
**Enjoy being together with your peers**				
	Yes	277 (77.6)	80 (22.4)	0.074**
	No	30 (90.9)	3 (9.1)	
**Friends influence decisions**				
	Yes	236 (75.6)	76 (24.4)	0.003*
	No	71 (91.0)	7 (9.0)	
**Accepted by peers**				
	Yes	282 (77.5)	82 (22.5)	0.025*
	No	25 (96.2)	1 (3.8)	
**Feel left out of things with peers**				
	Yes	196 (76.0)	62 (24.0)	0.064**
	No	111 (84.1)	21 (15.9)	
**Have more than 3 close friends in school**				
	Yes	290 (78.0)	82 (22.0)	0.095**
	No	17 (94.4)	1 (5.6)	
**Feel safe in school**				
	Yes	225 (75.0)	75 (25.0)	0.001*
	No	82 (91.1)	8 (8.9)	

* = Significant at p <0.05** = Significant at p < 0.10

**Table 3:** predictors of bullying perpetration among in-school adolescent in Sokoto metropolis, 2019 (n = 390)

Variables	cOR (95% CI)	aOR (95% CI)
**Age group** (10-14 years vs 15-19 years†)	0.61 (0.28 -1.00)	0.74 (0.40 - 1.39)
**Gender** (Male vs Female†)	2.53 (2.09 - 5.98)	2.70 (1.43 - 5.10)*
**Religion** (Christian vs Muslim†)	1.89 (1.46 - 5.70)	1.38 (0.42 - 4.53)
**Ethnic group** (Hausa/Fulani vs Yoruba/Ibo	0.36 (0.21 - 0.63)	0.43 (0.15 - 1.25)
**Family type** (Monogamous vs Polygamous†)	0.36 (0.21 - 0.63)	1.02 (0.28 - 3.74)
**School type** (Public vs Private†)	2.44 (1.49 - 4.00)	0.77 (0.38 - 1.55)
**Accommodation status** (Boarding vs day†)	6.25 (3.19 - 12.26)	7.93 (2.91 - 21.58)*
**Often experience parents quarrel or fight** (Yes vs No†)	5.17 (2.30 - 11.62)	5.23 (2.15 - 12.71)*
**Parents monitor where about and friends** (Yes vs No†)	3.80 (0.75 - 19.19)**	4.72 (0.59 - 37.86)
**Enjoy being together with peers** (Yes vs No†)	0.35 (0.10 - 1.16) **	1.71 (0.35 - 8.27)
**Friends influence decisions** (Yes vs No†)	0.31 (0.14 - 0.69)	0.77 (0.28 - 2.13)
**Accepted by peers** (Yes vs No†)	0.14 (0.02 - 1.03)	0.22 (0.03 - 1.93)
**Feel left out of things with peers** (Yes vs No†)	0.60 (0.35 - 1.03)	0.75 (0.38 - 1.47)
**Have more than three close friends in school** (Yes vs No†)	0.21 (0.03 - 1.59)**	0.26 (0.03 - 2.37)
**Feel safe in school** (Yes vs No†)	0.29 (0.14 - 0.63)	1.98 (0.68 - 5.73)

**†** = Reference group **aOR** = adjusted Odds ratio **CI** =Confidence Interval **cOR** = Crude Odds ratio*=Significant at p <0.05**Significant at p < 0.10

## Discussion

We observed that a high proportion of respondents had perpetrated at least one form of bullying. This high prevalence could be as a result of the believe parents and teachers have that bullying is part of experience Nigerian children should learn to tolerate as a process of growing up [28]. Although, there is increasing awareness about bullying now, this high proportion of bullying perpetration is worrisome considering the immediate and long-term effect bullying has on the educational achievement and social life of an individual. The perpetrator (bully) can be expelled from school, which in turn could trigger a lifestyle of hooliganism, cultism and financial dependence in the future. Furthermore, studies have shown that bullying perpetration at an early age could extend into adult life in the form of criminality, vandalism, sexual harassment and intimate partner violence [5,29]. For the victims, the consequences of bullying are far-reaching, ranging from academic problems to physical and mental illness. The high prevalence of bullying perpetration observed in this study is consistent with what was reported in previous studies in other parts (Benin and Port Harcourt) of Nigeria [20,21]. However, a lower prevalence has been reported the US and Canada [11,12,30,31]. The differences observed in this study could be as a result of attempts by schools and government to tackle bullying and the media attention given to bullying behavior in industrialized countries could be responsible for the difference. The most prevalent form of bullying perpetration observed in this study was verbal, closely followed by physical. This finding is consistent with previous studies in Nigeria and Israel [32,33]. However, some other studies in Nigeria [21,34] and the US [25] have found physical perpetration to be the most prevalent followed by verbal bullying. The high level of verbal bullying observed could be because it is the easiest to perpetrate and the most difficult to detect. Most times, no physical evidence to prove that a student is perpetrating verbal bullying. Therefore, this form of bullying can be on-going for a long time without being noticed by any teacher or the school authorities, leaving a significant impact on the health - especially the mental health and education of the victims.

Males were more likely to be perpetrators of bullying. The possible explanation for this could be that, in the study area, females are usually brought up to be shy and reserved. This finding could have an impact on future relationships, with the bullying perpetration manifesting in various forms such as intimate partner violence and other forms of domestic violence. The finding in this research is in line with what has been reported in earlier studies in Nigeria and US [34-36]. However, another study in the US found no gender difference in bullying perpetration [31]. The finding of no gender difference in the US study could be because boys and girls get equal treatment culturally. Being in boarding school was a significant predictor of bullying perpetration. This finding is not surprising as those who attend boarding schools spend lengthy periods of unsupervised time near their peers. The lengthy-time sets the stage for bullying that is usually not reported due to fear of reprisal and often no protection from school authorities. These factors can lead to normalization of bullying and hence the higher risk of bullying perpetration in boarding schools. This behaviour is unhealthy for mental development as the younger adolescents can take a cue from this, and the vicious circle of violence is maintained over generations of students. Growing up with this mentality could have severe, long-lasting consequences on the development of social skills among this group of adolescents. The finding in this study is in line with what was reported in Germany [37].

In this study, there was a significant association between bullying perpetration and conflicts between parents in the home. Those whose parents fight or quarrel often were more likely to be perpetrators of bullying. This unhealthy encounter presents a public health and safety issues in schools when adolescents imitate violent behavior exhibited by their parents at home. Similar findings were reported in previous studies in Zimbabwe and Italy [38,39]. These studies, including our research, were cross-sectional in design. However, there is consistency with the results of a longitudinal study in the United Kingdom, where factors like maltreatment and domestic violence were associated with bullying in school [40]. We recognize that this study has some limitations. Although the prevalence of bullying perpetration was high in this study, the fact that it was self-reported could have underestimated the prevalence because some bullies might not want to admit they perpetrate such. To limit this, we assured the students' confidentiality and anonymity before the commencement of data collection.

## Conclusion

In conclusion, there is a high prevalence of bullying perpetration among respondents with verbal and physical being the commonest forms. Those who often experienced parental conflicts and those in boarding schools are more likely to be perpetrators of bullying. School principals should pay attention to bullying behaviours among students, especially those in boarding schools; and parents should be educated on the consequences domestic violence can have on their children, especially outside the home front during parents and teachers association meetings.

### What is known about this topic

- Bullying extensively studied in America and Europe. It has been found to be the commonest form of violence in schools;
- Bullying perpetration is linked to a broad spectrum of adverse outcome for both the victim and the perpetrator;
- Bullying behavior in school has not gotten enough attention in Nigeria.

### What this study adds

- This study found a high prevalence of bullying perpetration in Sokoto metropolis;
- The commonest forms of bullying perpetrated in Sokoto metropolis is verbal bullying;
- Adolescents who often experience parental conflicts and who attend boarding schools are more likely to be bullies.
